# The Case of an Obstructed Stone at the Distal Urethra

**DOI:** 10.7759/cureus.1974

**Published:** 2017-12-20

**Authors:** Kelcy Higa, Stephen Irving, Richard J Cervantes, Jayce Pangilinan, Laura R Slykhouse, Dale P Woolridge, Richard Amini

**Affiliations:** 1 Department of Emergency Medicine, University of Arizona; 2 College of Medicine, University of Arizona College of Medicine-Tucson

**Keywords:** urethral stone, urinary calculi, point-of-care ultrasound, pocus

## Abstract

This report highlights a presentation of urinary calculus impacted at the urethral meatus and bedside extraction after evaluation with point-of-care ultrasound (POCUS). Visualization of a stone at the urethral meatus prompted a point-of-care ultrasound of the penile shaft and glans. The ultrasound ruled out anatomic variations such as urethral diverticula and as a result bedside removal was expedited. The stone was successfully removed with traction and intraurethral lidocaine gel without urethral lesions or injury to the meatus. Bedside ultrasound is readily available in the emergency department and can be used to characterize urethral foreign bodies, evaluate urethral anatomy, and assess the likelihood of bedside removal.

## Introduction

Urinary calculi are commonly treated in the emergency department (ED). In 2009 alone, there were roughly 1.3 million ED visits (approximately 3,600 every day) related to kidney stone disease [1]. Urethral stones, however, are rarely encountered in the ED and account for fewer than 1% of all kidney stones [2]. They can be migrant or native and often present as urinary retention, perineal and rectal pain, or external meatus and urethral pain [3-4]. Migrant stones develop in the kidney or bladder and migrate into the urethra [3]. Native urethral stones form in the urethra and are associated with strictures, urethral diverticula, chronic infection, and urethral foreign bodies. Diagnostic imaging methods in the ED for urethral stones include radiograph of the abdomen and pelvis or retrograde urethrogram [4]. Although literature describes cases of impacted urethral stones, few cases demonstrate instances where point-of-care ultrasound (POCUS) was used to better evaluate an impacted urethral stone.

## Case presentation

**Figure 1:**
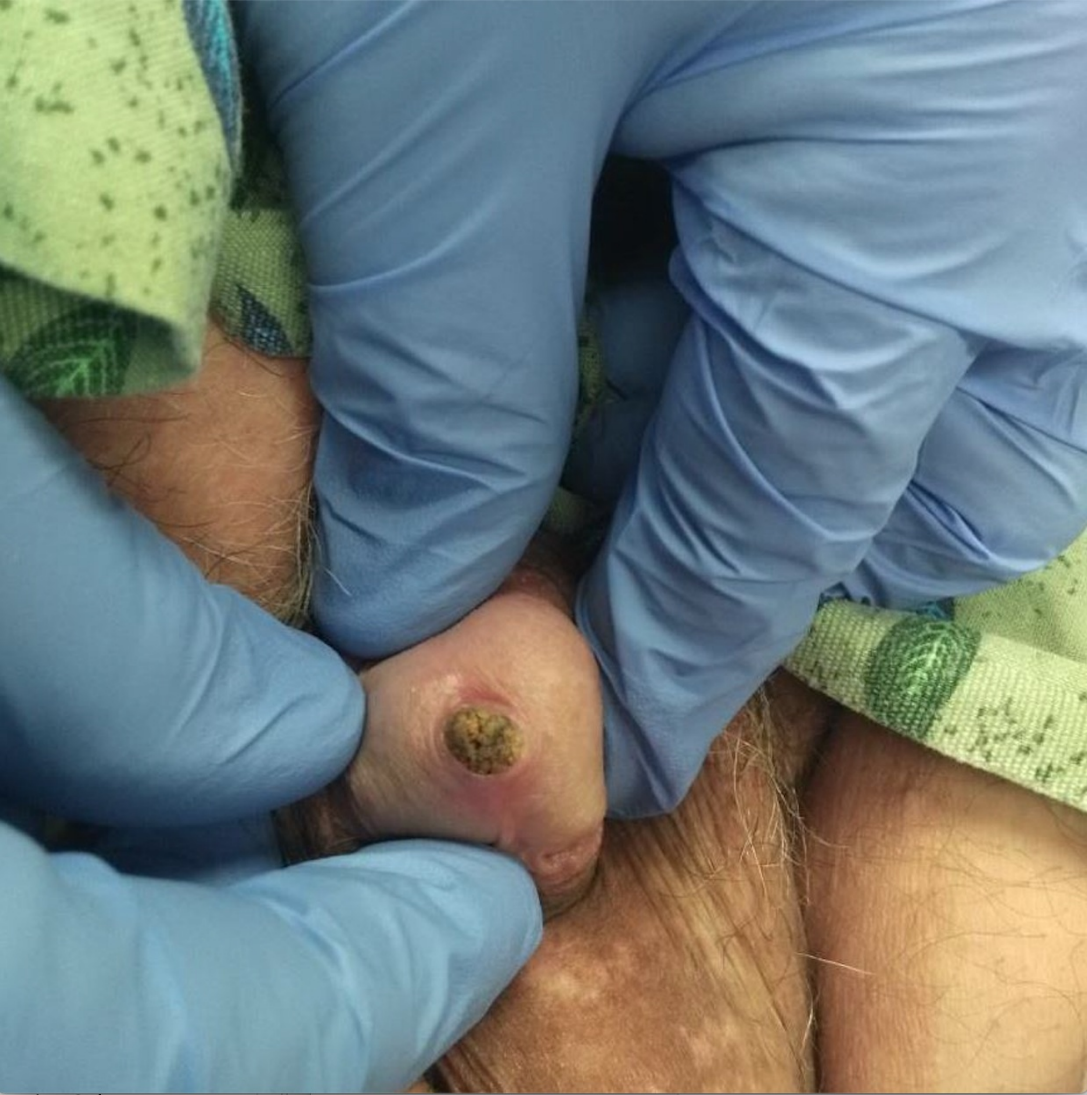
Stone obstructed at the distal urethra Physical examination revealed a stone obstructing the distal urethra.

A 57-year-old man presented to the emergency department (ED) with left-sided flank pain for the past 72 hours. His past medical history was significant for left-sided renal stones, and his presentation was similar to previous episodes of renal colic. On arrival to the ED, his left flank pain had resolved; however, he developed distal penile pain and pressure. He was unable to urinate and noted a hard mass at the tip of his penis. On examination, a small circular foreign body, approximately 5 mm, appeared to be impacted at the urethral meatus (Figure 1).

**Figure 2:**
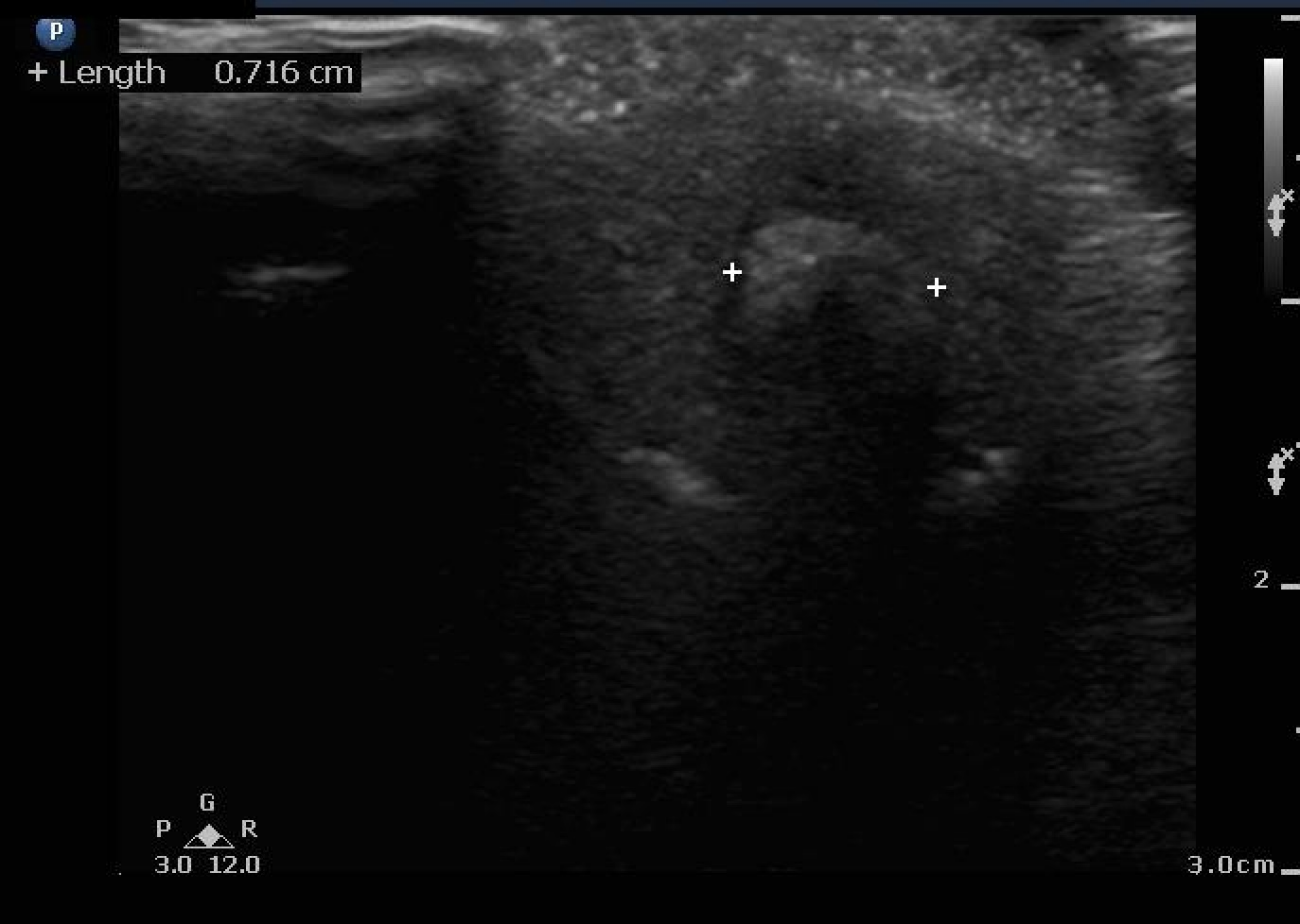
Transverse urethra with impacted stone

A bedside ultrasound was performed with a high frequency linear array probe. It was placed along the short axis of the penile shaft and glans demonstrating a hyperechoic, circular structure with posterior acoustic shadowing (Figure 2).

**Figure 3:**
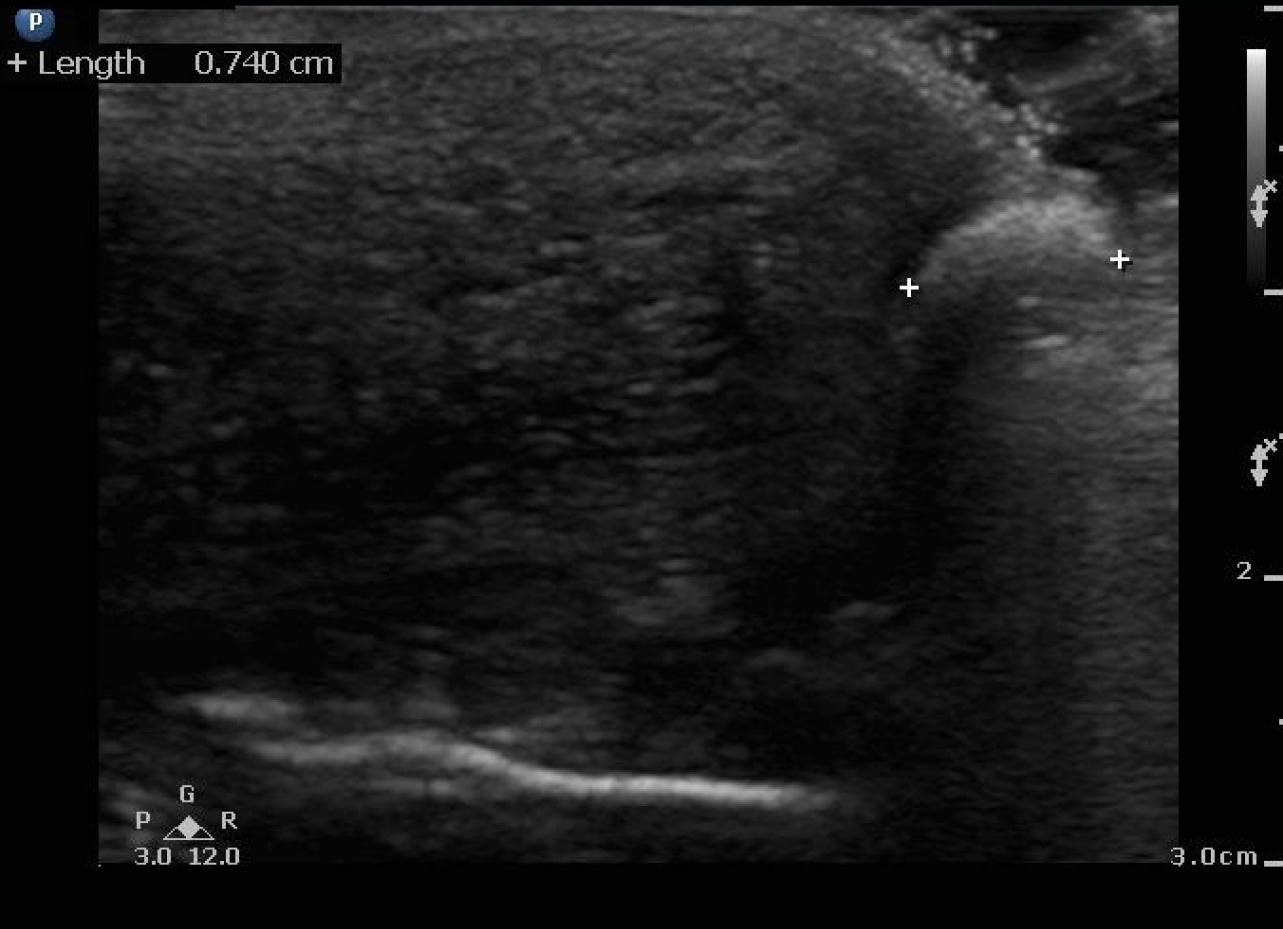
Sagittal urethra with impacted stone

The ultrasound was also placed along the long axis of the penile shaft and glans demonstrating a hyperechoic, circular structure with posterior acoustic shadowing (Figure 3).

The structure was measured to be 7.2 mm x 7.4 mm and was impacted at the urethral meatus. On color flow, there was a twinkle artifact (Video 1).

**Video 1:** Twinkle artifact

**Figure 4:**
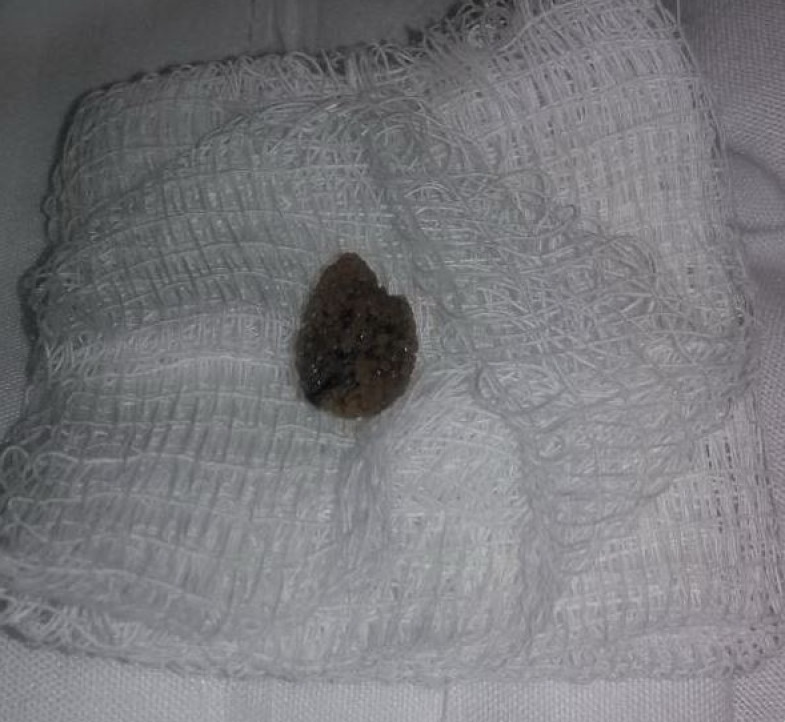
Urethral stone after removal

There was no evidence of urethral distention, urethral diverticula, or other foreign bodies. Based on the ultrasound imaging, we felt that the stone could be successfully removed with manipulation of the urethral meatus. An 11 mm x 8 mm stone (Figure 4) was removed with traction and application of intraurethral lidocaine gel. There was no evidence of urethral lesions or injury to the meatus upon removal.

## Discussion

In most cases of penile urethral calculi, a stone is palpable along the expected urethral course on examination. Nearly 100% of urethral calculi are radiopaque and can be visualized with plain radiographs [4]. Retrograde urethrogram or cross sectional imaging can also be used to aid in diagnosis; however, these procedures can be painful and expose the patient to more ionizing radiation. Due to visualization of the stone at the urethra meatus, we opted for bedside ultrasound for rapid evaluation. Using ultrasound, the penis can be visualized circumferentially, allowing calculi to be assessed in multiple planes to determine the size and location in the urethra [5].

POCUS has become an integral part of emergency medicine (EM) residency training and has many applications as a diagnostic tool. Renal ultrasound is a component of POCUS training and assessment; most EM graduates will have significant experience using ultrasound at the bedside [6-7]. POCUS is available in all academic EDs and most community EDs and has been shown to be effective in urinary tract imaging as a quick and effective way to locate and characterize distal urethral stones to assess for obstruction [8]. The use of POCUS allows for a more rapid diagnosis, shorter length of stay, and decreased exposure to radiation by decreasing the need for computed tomography [7].

In the case above, we used ultrasound to assess the size of the urethral stone as well as evaluate the patient for urethral anatomic variations such as urethral diverticula. The presence of these anatomic variations would have made manual removal of the stone more complicated and would have prompted a urology consult [9]. For stones in the anterior urethra, “milking” has been reported to be successful in several case reports [3]. For calculi that cannot be removed with simple manipulation, stone extraction can be performed by urethrotomy [4] or in-situ lithotripsy [4]. An acutely impacted urethral stone can lead to severe pain, urinary retention, urethral injury, and obstructive renal failure. Stones that are undiagnosed for an extended period of time may cause incontinence, impotence, urethrocutaneous fistulas, and post-obstructive renal failure [4]. This indicates the importance of a timely diagnosis and treatment in the ED.

Our case demonstrated how POCUS at the bedside allowed for prompt diagnosis of the urethral calculi and accelerated removal of the stone. However, the size of the stone differed significantly from our ultrasound measurements. This may partly be due to not assessing the vertical axis. We measured the stone in only two planes when three would have assessed for the true size. Additionally, bladder and renal ultrasound should have been obtained to evaluate for additional stones and complications secondary to urethral stone impaction. In the ED, we suggest the use of POCUS when urethral stones are suspected. The clinician can ultrasound the urethra in transverse and longitudinal imaging along the shaft of the penis. Given the relatively shallow depth required to successfully ultrasound the shaft of the penis, readily available high frequency linear transducers are sufficient to achieve quality imaging.

Sending urethral calculi for pathology analysis can help in identifying the origin of the stone. Struvite stones predominate in primary urethral calculi [3], while calcium oxalate is the predominate component in migratory urethral stones [4]. Patients with urethral calculi should follow up with the urology department, as urethral calculi can be associated with anatomic variations such as urethral diverticula or stricture.

## Conclusions

POCUS is readily available in the ED and can be used as an adjunct imaging modality to characterize urethral foreign bodies, evaluate urethral anatomy, and assess the likelihood of bedside removal.
